# Hysterical Muscular Contractions

**Published:** 1880-11

**Authors:** W. T. Speaker

**Affiliations:** Mount Morris, Ill.


### ARTICLE IV.

### HYSTERICAL MUSCULAR CONTRACTIONS. By W. T. SPEAKER, M.D.

There is scarcely a muscle or group of muscles in the body which may not become the seat of muscular contraction; this fact has long been known to the profession, but resemblance to organic disease is such that physicians and surgeons are still deceived by its counterfeits. Hysterical conditions of the muscular coats of the intestines are of most frequent occurrence, in producing great pain and nervous disturbances. I have met several cases in my practice, of hysterical muscular contraction, which makes its appearance in the form of a tumor. The several which I have seen were in the flat muscles of the abdomen, and in either case they were not accompanied with pain. They had remained a considerable length of time without any treatment; no enlargement or diminution was observed, but under full doses of the bromides they rapidly disappeared.

Spasmodic stricture of the urethra, caused by intense emotional excitement and accompanied by various hysterical phenomena, is so commonly met with in ordinary practice that it scarcely escapes the attention of the physician or surgeon. The diagnosis of these cases is not very difficult to obtain, if close attention is paid to the clinical history of the patient. It will generally be found that there have been other indications of the hysterical conditions present; that these contractions have made their appearance at other times and, like "phantom tumors," have duly disappeared. One case which I have had under my care the last year, is that of a woman thirty-five years old, married, with two children. This lady has frequently called my attention to a tumor situated in the posterior part of the latissimus dorsi muscle. Several times I have noticed an intense contraction of the muscle, at other times none whatever. These contractions seem to be produced by a power of the will or from a diseased functional disorder of the brain. Another case is that of a girl thirteen years of age. When first called to see her, my observation was that it was a case of wry neck, but upon a close diagnosis I discovered an intense contraction of the right sterno-cleido-mastoid muscle, and the head was drawn violently in the direction opposite to that which had formerly existed. The affection had developed suddenly, so far as she knew, without obvious cause, and was unaccompanied with pain. Her general health up to this time had been good. The treatment consisted of plenty of fresh air, occasional drives, and cold baths. Medically the bromide of zinc proved efficient in relaxing the tension of the muscle. The formula was as follows: ℞. Zinci bromid. ʒij, aquæ dist. ℥ij. M. S. Ten drops three times a day, increasing a drop at a dose for each day. I increased the dose until she was taking about forty drops three times a day.

Another case was that of a young married woman. I was called in early one morning and found her laboring with what she presumed to be a miscarriage. The symptoms, on entering the room, led me to think she was in the third stage of labor, with the pains coming on from five to ten minutes. Upon examination I did not observe any dilatation of the os uteri, nor the least contraction of the uterus, but severe contractions of the abdominal muscles. I watched the case closely for nearly an hour and left, fully convinced that it was purely hysterical. I gave full doses of bromide of potassium, and in less than twenty-four hours all symptoms had subsided, and there was no further trouble until confinement.

About one year ago I was invited to see a young lady sixteen years of age, in company with Dr. B., a neighboring physician, who informed me that the lady was suffering from an atrophy of the muscles of the right leg. She had been under treatment for about one year for this trouble, but without any perceptible improvement. There was a slight contraction of the flexors of the leg, and the atrophy gave the appearance of an enlargement of the knee-joint, which, however, I was confident did not exist. Although the patient declared that she could not straighten the limb, and that every effort to do so gave her great pain, I seized the leg with one hand and the thigh with the other and forcibly extended the knee-joint. There was no great degree of pain produced, and though the flexed position was again assumed, I was confident that by proper treatment the limb could be restored to its normal condition. Faradization was now applied for the benefit of the atrophied muscles; the joint was repeatedly flexed by passive motion, the muscles were kneaded for a half-hour morning and evening, and she was encouraged to use the member by walking on it and flexing it by voluntary power. She drove out every pleasant day in the pure air, and was allowed to participate in any light and enjoyable amusement. The free use of the bromide of sodium was given with the bitter tonics. These means were entirely successful, and a year has now passed and she is entirely free from any hysterical trouble whatever.

Hysteria in all its varied aspects is usually recognized without any difficulty. A few isolated symptoms of the disease will suffice to reveal its nature to the practiced eye. The object of this paper has been to call attention to a class of disorders liable to be mistaken for others of a more serious import, and to the fact that recovery is frequently the apparent result of emotional disturbance. Among the physical influences, those of a depressing nature have the most marked effect upon producing or developing this disease. Thus in the treatment if we can bring the mind to bear upon this disease, and produce a different degree of thought and feeling, a cure is often accomplished. Therefore it is easy to see why the great army of mesmerizers, magnetizers, magicians, spiritualist mediums and quacks produce such an influence upon their patients. It is generally implied that these phenomena are of merely a functional, subjective character, more or less dependent on the state of the mind, more especially the will, and that a change of the mental condition has been naturally followed by a change in the phenomena, although apparently physical. Such is the broad definition of the imagination, as it presents itself to the mind when employed in reference to medical facts of every day occurrence. It matters little to the patient by what name the remedy is called, whether "imagination" or some of the "pathies" of the day; but to the philosophical practitioner it ought to matter a great deal; it ought to be a question of exceeding interest.

MOUNT MORRIS, ILL., Sept. 30, 1880.

---

PARIS HOSPITALS.—The Paris correspondent of the *Lancet* says: Now that public attention has been forcibly called to the insanitary condition of the town generally, it would be well if some influential organ would protest against the disgraceful condition of several of the Paris hospitals. The Hôpital Laennec, for instance, is very little better than a slaughter-house for incurables. The wards situated upon the ground-floor are especially badly kept. Odors which emanate from the closets, impregnate the atmosphere and culiminate in an almost insupportable stink. Close to the small dungeon which is used as a speculum-room, a few days since I noticed, with surprise, on the clothes of a woman some lively specimens of the *Cimex lectularius*, and on expressing my astonishment to one of the pupils he informed me that the insect has its habitat at Laennec. He furthermore volunteered the statement, which is probably strictly true, that a few months' residence at that institution is sufficient to tuberculize the strongest inmate.—*Louisville Medical News.*

PRESERVATION OF THE COLORS OF DRIED PLANTS.—M. Stoebzl states that this is most effectually done by passing the plant slowly through a solution of one part of salicylic acid in six hundred parts of alcohol, heated to boiling point in an evaporating vessel (*Journal de Pharmacie*). The superfluous liquid is then to be shaken off, and the plant placed between blotting-paper and pressed as usual. The blotting-paper must be frequently renewed, especially at first. Plants so treated dry rapidly, and preserve their natural colors more perfectly than under any other procedure.—*Louisville Medical News.*

